# Changes in human skull bone marrow during pregnancy and postpartum: an exploratory case report

**DOI:** 10.1186/s12884-026-08969-7

**Published:** 2026-03-20

**Authors:** Polona Kalc, Eileen Luders, Robert Dahnke

**Affiliations:** 1 Department of Neurology, Jena University Hospital, Jena, Germany; 2 Department of Psychology, University of Deusto, Bilbao, Spain; 3 IKERBASQUE, Basque Foundation for Science, Bilbao, Spain; 4 Department of Women’s and Children’s Health, Uppsala University, Uppsala, Sweden; 5 School of Psychology, University of Auckland, Auckland, New Zealand; 6 Laboratory of Neuro Imaging, School of Medicine, University of Southern California, Los Angeles, USA

**Keywords:** Skull, MRI, Pregnancy, Postpartum, Bone marrow adipose tissue

## Abstract

**Background:**

The physiology of bone marrow adipose tissue during pregnancy and lactation remains poorly understood.

**Case presentation:**

In this exploratory single-subject magnetic resonance imaging study, we investigated the intensity of skull bone marrow before, during and after pregnancy of a 38-year-old primiparous woman.

**Conclusion:**

We observed a curvilinear pattern of change. These preliminary findings require validation using quantitative fat imaging in larger cohorts and highlight the need for further research into maternal bone health during pregnancy and lactation.

**Supplementary Information:**

The online version contains supplementary material available at 10.1186/s12884-026-08969-7.

## Background

Maternal skeletal homeostasis in humans is under considerable strain during pregnancy and lactation, with high bone turnover and increased bone resorption [1–3]. More specifically, existing studies report a pronounced decrease in bone mineral density (BMD), particularly in areas rich in trabecular bone, such as the lumbar spine and the trochanteric region [4–8]. However, this decrease is transient and progressively reversed in the post-weaning period [1, 9]. With respect to bone marrow adipose tissue (BMAT), murine studies have demonstrated decreases during postpartum and lactation [10, 11]. However, even in animal models, information on changes in BMAT during pregnancy is lacking, and data on changes in BMAT in humans spanning pregnancy and postpartum are entirely absent [2].

BMD and BMAT are crucial factors in the context of pregnancy and the postpartum period, as they capture complementary aspects of skeletal remodeling and energy metabolism under conditions of heightened physiological demand. While some information on BMD in humans exists, there is a lack of data with respect to BMAT. In the present study, we leveraged an existing densely-sampled dataset of a single woman, who underwent longitudinal brain magnetic resonance imaging (MRI) before, during, and after pregnancy.

## Presentation of the case and the results

The study participant was a 38-year-old primiparous woman, who underwent in vitro fertilisation treatment to achieve pregnancy. This enabled precise monitoring of conception and gestation week. The pregnancy was uneventful, and delivery occurred at full term through vaginal birth [12]. The current study was based on structural images of the head from the aforementioned woman, as collected in the framework of the Maternal Brain Project [12]. More specifically, T1-weighted MR data were acquired at the University of California, Santa Barbara and Irvine on 3 T Prisma scanners with a 64-channel phased-array head/neck coil and a magnetization prepared rapid gradient echo (MPRAGE), using the following parameters: repetition time (TR) = 2,500 ms, time to echo (TE) = 2.31 ms, inversion time (TI) = 934 ms, flip angle = 7°, and 0.8 mm thickness. Importantly, the decision to use an MPRAGE imaging sequence in this exploratory study was solely based on data availability (i.e., MPRAGE is not a standard sequence for quantifying adipose tissue). The entire image series consisted of 26 scans taken longitudinally covering a pre-conception period over two months (*n =* 4), pregnancy over 9 months (first trimester: *n =* 4; second trimester: *n =* 6; and third trimester: *n =* 5), and a postpartum period over two years (*n =* 7). Note, the final set of brain scans was 25 as one scan needed to be removed (see the Method section). The Maternal Brain Project study was approved by the Human Subjects Committee of the University of California, Irvine (UCI), and the participant gave her written informed consent.

**Fig. 1:**
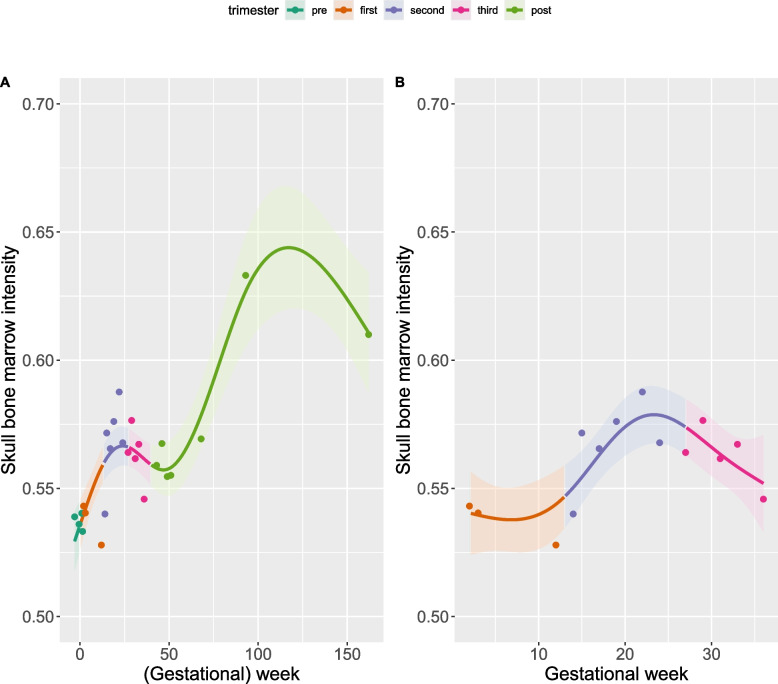
Changes in skull bone marrow intensity between preconception and postpartum (**A**), and during pregnancy only (**B**). The colored regression lines and dots correspond to the 25 longitudinal measurements: 4 times during pre-conception over 2 months (dark green); 14 times during pregnancy over 9 months (3 times in the first trimester [orange]; 6 times in the second trimester [blue]; 5 times in the third trimester [pink]), and 7 times during postpartum over two years (light green)

Figure 1 shows descriptive results of this single-case exploratory study. We demonstrated that there are measurable changes in the intensity of skull bone marrow throughout pregnancy and postpartum in a primiparous woman, potentially implying changes in BMD and bone marrow adiposity. As the study was based on a single participant and the measures were not derived from quantitative MRI imaging, the results should not be overgeneralised.

## Discussion and conclusion

Our preliminary results indicate modest increases in skull bone marrow intensity during the first trimester of pregnancy, followed by mild increases in the second trimester, and mild decreases during the third trimester. There is an inverse relationship between BMD and BMAT, as observed outside the framework of pregnancy [13–16]. An increase in skull bone marrow intensity could be associated with an increase in bone marrow adiposity and a decrease in BMD. Previous studies have reported little or no decrease of BMD after pregnancy [5], which would potentially correspond to little or no increase of skull bone marrow intensity. However, there were noticeable increases in skull bone marrow intensity throughout the duration of breastfeeding (before week ~120). Further studies examining BMAT volume are warranted, also given that animal studies reported a decrease in bone marrow adipocyte volume during lactation [11]. Nevertheless, the observed increase in our study could potentially reflect delayed effects resulting from the increased metabolism towards the end of pregnancy [17], and/or a loss of bone mass due to lactation, as previously reported [18, 19]. Of note, we observed a decrease in skull bone marrow intensity later on (after week ~120) during the post-lactation period. Interestingly, overall, changes during postpartum were more pronounced than during pregnancy.

Limitations of our study include a sample size of n=1, which means that the observed variations across pregnancy and postpartum could also reflect changes related to idiosyncratic lifestyle factors, either instead or in addition to potential biological changes due to pregnancy and lactation. Moreover, we estimated the intensity of the bone marrow adipose tissue, which is a qualitative rather than a quantitative measure and thus less suitable to the study of adipose tissue. Follow-up research might benefit from investigating (skull) bone marrow adipose tissue properties using refined MRI techniques, such as Dixon imaging or MRI proton density fat fraction. Our study was based on T1-weighted images of the head, which were optimised for measuring brain properties [12]. In contrast, the majority of existing studies have used DXA measurements to assess BMD before and after pregnancy [4–9], or employed heel bone broadband ultrasound attenuation and/or molecular bone turnover markers during each trimester to minimise radiation exposure (e.g., refs [4, 8]). Last but not least, skull bone marrow intensities can be affected by acquisition-related variability, although it was ensured that image acquisition protocols, software, and model were identical across time points and scanners. Given the aforementioned limitations, the biological interpretability of the current study is limited. Therefore, further studies are required to draw valid conclusions regarding the effects of pregnancy and lactation on BMAT in humans.

To summarize, this is the first report of longitudinal variations in skull bone marrow signal intensity on routine T1-weighted MRI during pregnancy and lactation in a human participant. Our findings provide novel insights into an underexplored area of research, particularly given the integral role of bone marrow adipose tissue in energy metabolism during energetically demanding states. Despite its limitations, our findings provide preliminary evidence that supports the feasibility and potential relevance of examining bone marrow adiposity across this special period in a woman’s life. A better understanding of bone marrow dynamics during pregnancy and the postpartum period might also be particularly relevant in the context of pregnancy- and lactation-associated osteoporosis, as well as metabolic conditions such as gestational diabetes. Of note, our study focussed on bone marrow adiposity of the skull in particular, which generally receives little attention in comparison with other skeletal sites as it is not the most clinically relevant site for osteoporosis. However, the skull may be a sensible target for investigation in the framework of pregnancy, as it is largely unaffected by the mechanical load. In other words, any changes in its structure largely occur without any confounding effects of weight-bearing [20]. This is especially relevant as changes in weight-bearing are a phenomenon of pregnancy as a result of both maternal tissue accretion and fetal growth [21].

## Method

### Image processing

The T1-weighted images were processed using Statistical Parametric Mapping (SPM12; http://fil.ion.ucl.ac.uk/spm/) in MATLAB 2021a (MathWorks, Natick, Massachusetts, United States) and running the expert mode of the Boney Toolbox (https://github.com/robdahn/boney), as previously described in ref [22]. Briefly, after extracting head tissue classes and segmenting bone regions pertaining to the skull (i.e., frontal, occipital, left and right parietal, left and right temporal bone), any incorrectly assigned segments were automatically corrected. The intensities within the bone were normalized based on the average intensity of the white matter. Then, an intensity-derived skull bone marrow metric was calculated as the weighted average of the signal intensities of the corrected skull segment between inner and outer cortical tables. The bone marrow intensity value pertaining to session 7 (first trimester) was removed as the scan had a different inhomogeneity field compared to the other 25 scans.

### Statistical analysis

All analyses and visualisations were implemented in RStudio, version 2024.12.1+563 (RRID:SCR_000432) using the packages *mgcv,* version 1.9–4.9 (ref [23]) and *ggplot2,* version 4.0.0 (ref [24]; RRID:SCR_014601). Due to the sample size of 1, we do not report inferential statistics.

### Main analyses

Two main descriptive analyses were conducted, one with respect to the time frame between pre-conception and postpartum (25 time points; Analysis I) and one pertaining to pregnancy only (14 time points; Analysis II), as subsequently detailed.

Analysis I: A generalised additive model (GAM) was fitted to the 25 measurements of bone marrow intensity (cubic spline basis, k = 5, smoothing = GCV). The basis dimension (k = 4–5) was evaluated using *gam.check* function. While k = 4 suggested a linear trend (edf = 1, k-index = 0.78, *p*-value = 0.14), this solution seemed to be slightly underfitted. We thus selected k = 5 (edf = 3.92, k-index = 0.82, *p*-value = 0.13) which yielded a more normal distribution of residuals.

Analysis II: A GAM was fitted to the 14 measurements of bone marrow intensity during pregnancy only (cubic spline basis, k = 4, smoothing = GCV). Evaluation of basis dimension supported k = 4 (edf = 2.76, k-index = 0.96, *p* = 0.3) with improved normal distribution of residuals.

### Supplementary analyses

Finally, given that data were acquired on two scanners (both identical in type and software, as well as following the same imaging protocol), the aforementioned analyses were repeated while restricting the dataset to pregnancy and postpartum measures obtained on the same scanner.

More specifically, supplementary analysis I was conducted using measures from the following time points: first trimester (*n =* 1), second trimester (*n =* 6), third trimester (*n =* 5), and postpartum (*n =* 7). Fitting a GAM to these 19 data points (cubic spline basis, k = 4, smoothing = GCV), revealed a linear trend, as shown in Figure 1S A (Supplementary Material).

Supplementary analysis II was conducted using measures from the following time points: first trimester (*n =* 1), second trimester (*n =* 6), and third trimester (*n =* 5). Fitting a GAM to these 12 data points (cubic spline basis, k = 4, smoothing = GCV) demonstrated a nonlinear trend, as shown in Figure 1S B (Supplementary Material).

Importantly, the outcomes of the supplementary analyses (reduced data set) remained relatively stable in comparison to the main analysis (full data set). The linear pattern in the reduced data set, contrasting the curvilinear pattern in the full data set, is likely due to the reduced number of data points (see Figure 1S; Supplementary Material).

## Supplementary Information

Supplementary Material 1.

## Data Availability

The used data is publicly available at [https://openneuro.org/datasets/ds005299]. The code used to derive these findings is available at GitHub: [https://github.com/robdahn/boney].

## Abbreviations

BMAT: Bone marrow adipose tissue
BMD: Bone mineral density
DXA: Dual-energy X-ray Absorptiometry
GAM: Generalised additive model
GCV: Generalised-cross validation
MR: Magnetic resonance
