# Supplementary material for: Changes in human skull bone marrow during pregnancy and postpartum: an exploratory case report
Source: BMC Pregnancy Childbirth. 2026 Mar 20;26:387. doi: 10.1186/s12884-026-08969-7 (PMC13064234; doi:10.1186/s12884-026-08969-7)

Figure S1. Change in skull bone marrow based on data from only one scanner during the whole measurement period **(A)** and during gestation only **(B)**.

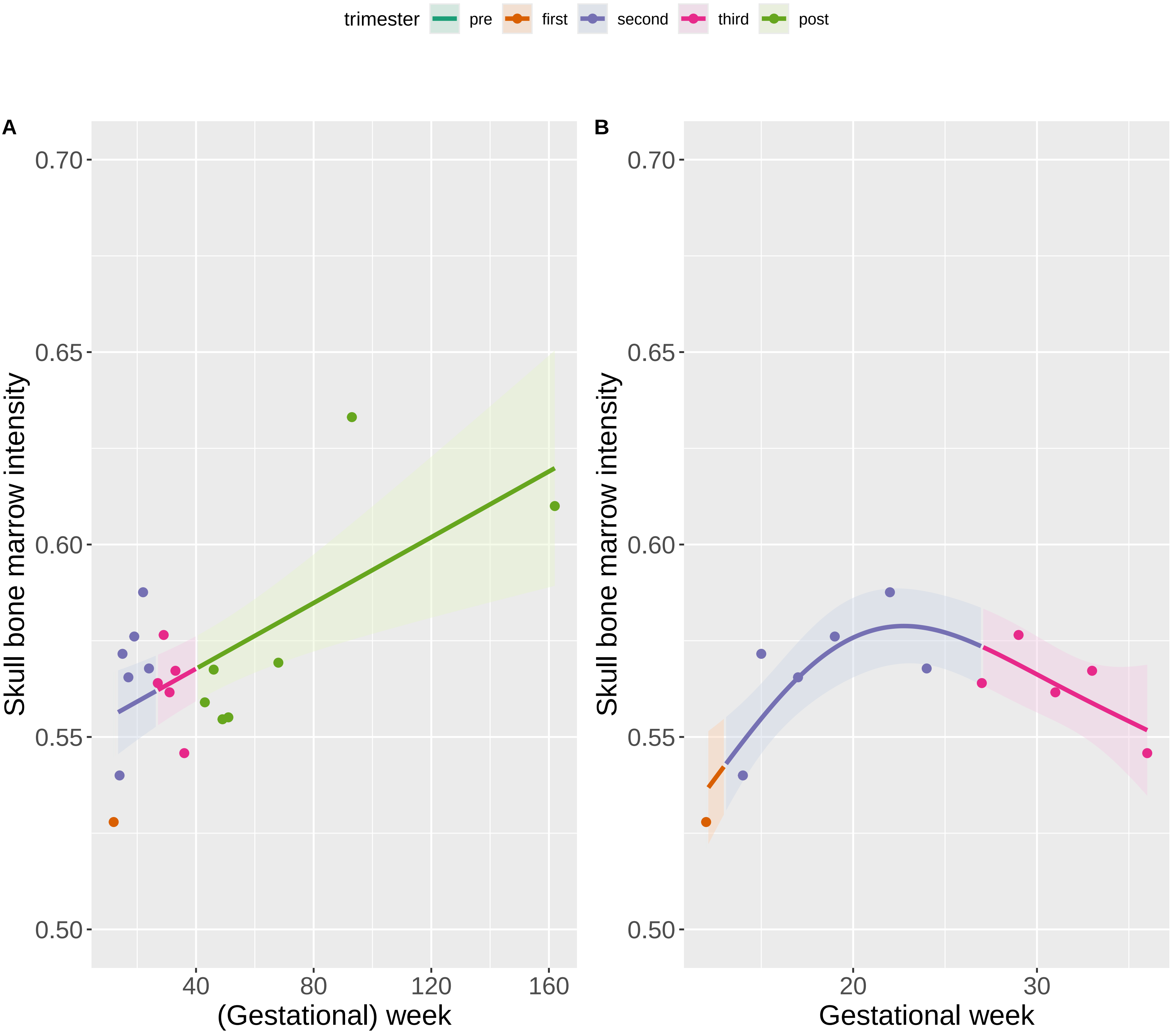

Supplement: Supplementary file 1 — Supplementary Material 1. [file 12884_2026_8969_MOESM1_ESM.pdf]
